# Effects of Treatment with L-Thyroxine on Chronic Urticaria in Subjects with Autoimmune Thyroiditis: A Systematic Review and Meta-Analysis

**DOI:** 10.3390/ijms27146348

**Published:** 2026-07-17

**Authors:** Monia Bordoni, Alessandro Ciarloni, Nairus Aboud, Elisabetta Sidori, Francesca Silvetti, Augusto Taccaliti, Alberto Vassallo, Marilina Romeo, Gianmaria Salvio, Giancarlo Balercia

**Affiliations:** 1 Endocrinology Clinic, Department of Clinical and Molecular Sciences, Polytechnic University of Marche, 60126 Ancona, Italy; m.bordoni@pm.univpm.it (M.B.); a.ciarloni@pm.univpm.it (A.C.); n.aboud@pm.univpm.it (N.A.); e.sidori@pm.univpm.it (E.S.); francesca.silvetti@ospedaliriuniti.marche.it (F.S.); a.taccaliti@staff.univpm.it (A.T.); g.balercia@staff.univpm.it (G.B.); 2 Department of Diabetology and Endocrinology, Ospedale Maggiore di Lodi, 26900 Lodi, Italy; alberto.vassallo@asst-lodi.it; 3 Endocrinology and Diabetes Unit, Humanitas Gavazzeni, 24125 Bergamo, Italy

**Keywords:** Hashimoto’s thyroiditis, L-T4, hypothyroidism, chronic urticaria, autoimmunity, thyroid peroxidase

## Abstract

Chronic urticaria (CU) is a condition characterized by recurrent wheals/angioedema lasting longer than 6 weeks. It is treated in a stepwise manner, starting with antihistamines and escalating to more advanced therapies, if needed. It is frequently associated with autoimmune thyroiditis (AT), and thyroid autoantibodies and thyroid-stimulating hormone (TSH) may contribute to the severity of symptoms, though the exact pathogenesis remains unclear. We performed a systematic search on Scopus and Pubmed databases, finding nine studies published between 1989 and 2018. Five studies were therefore included in our meta-analysis, and we found that the treatment with L-thyroxine (LT4) is associated with a significant improvement in CU symptoms (sMD −2.31, 95% CI −2.98 to −1.64; *p* < 0.00001). However, when compared with placebo or standard care, LT4 did not demonstrate superiority in terms of symptom scores. It is unclear whether it provides greater benefit in individuals with normal thyroid function or hypothyroidism, or whether it is more effective when used as suppressive or replacement therapy. Therefore, evidence for LT4 treatment improving urticaria is conflicting, with uncertain benefit prompting further evaluation and in-depth study of the molecular mechanisms and markers linking CU and AT.

## 1. Introduction

Chronic urticaria (CU) is a mast cell-driven disease characterized by the development of wheals, angioedema, or both, lasting 6 weeks or more [1].

The aim of treatment is disease control with complete resolution of signs and symptoms. Current guidelines recommend a stepwise approach to management; the first step consists of daily administration of second-generation H1 antihistamines (e.g., cetirizine, fexofenadine, loratadine, desloratadine, levocetirizine, bilastine, rupatadine, or ebastine). However, complete disease control is rare with standard doses, and dose escalation and adjunctive therapies are often needed, including H2-antihistamines or leukotriene receptor antagonists. Short-term systemic corticosteroids (20–50 mg/day for <10 days) may also be used for acute exacerbations. Omalizumab and cyclosporine are used as a last resort in unresponsive patients [2].

In the last 40 years, an increasing number of reports have suggested a link between CU and autoimmune diseases, including rheumatoid arthritis, systemic lupus erythematosus, Sjogren’s syndrome, and type 1 diabetes mellitus [3].

The association between autoimmune thyroiditis (AT) and CU is well-established, with 10–28% of CU patients also having autoimmune thyroid disease (most commonly Hashimoto’s thyroiditis) [2]. Hashimoto’s thyroiditis is the most common cause of acquired hypothyroidism, and the current standard of care for this condition is replacement therapy with L-thyroxine (LT4) [4]. Patients with coexisting AT are also known to suffer from more severe and long-lasting forms of CU, compared with subjects without thyroid autoimmunity [5].

Patients with CU typically exhibit higher levels of IgG anti-thyroid antibodies compared with controls [6]. The role of thyroid autoantibodies in the pathogenesis of CU, however, is still poorly understood; indeed, there is no demonstrated epitopic cross-reactivity between thyroid autoantibodies and autoantibodies related to CU [7].

Nevertheless, a dual pathogenic model in which IgE- and IgG-mediated mechanisms are responsible for mast cells activation and urticarial symptoms have been proposed. Patients with thyroid autoimmunity produce anti-thyroid peroxidase (TPO) and anti-thyroglobulin IgE antibodies, which bind to the high-affinity IgE receptor on mast cells and basophils. When the circulating autoantigen cross-links these IgE antibodies, it triggers basophil activation and mast cell degranulation, thus releasing histamine and other inflammatory mediators that cause wheals and angioedema [8]. Anti-TPO IgG autoantibodies, instead, can directly activate mast cells via surface receptors or by binding to IgE itself [7]. IgG autoantibodies are believed to generate an inflammatory response in the thyroid gland which leads to a generalized inflammatory state that increases the overall sensitivity of mast cells to other stimuli [9]. This low-grade inflammatory state may produce thyroid protein immune complexes that could activate the classical complement pathway, with the generation of C3a and C5a, and subsequent triggering of mast cells and basophils in patients with CU [10]. Interestingly, Aversano et al. also proposed a key immunomodulatory role for thyroid-stimulating hormone (TSH); indeed, they hypothesize that TSH could have a cytokine-like activity and may interact with cells of the immune system, thus leading to the release of more cytokines and interleukins which directly cause an inflammatory state of target organs such as the skin. In this context, suppression of TSH is suggested as a potential mechanism for lowering the inflammatory response [11]. However, the evidence supporting LT4 treatment for improving CU symptoms in patients with thyroid autoimmunity is conflicting.

In this systematic review, we aim at evaluating the effects of LT4 on CU in patients with autoimmune thyroid disease.

## 2. Materials and Methods

This study was conducted following the guidelines of the Preferred Reporting Items for Systematic reviews and Meta-Analyses (PRISMA) statement (Table S1) [12]. The review was registered on PROSPERO (https://www.crd.york.ac.uk/prospero/ (accessed on 25 August 2025)) with number CRD420251133491.

### 2.1. Search Strategy

A systematic search was conducted through Scopus and PubMed databases to identify any relevant literature published up to October 2025. The following terms were combined: “Chronic”, “Urticaria”, “Hashimoto”, “Thyroiditis”, “Thyroxine”, and “Levothyroxine”. The search was conducted independently by 2 authors (G.S. and A.C.), and disagreements were resolved through discussion with a third author (G.B.).

### 2.2. Selection Criteria

The eligible studies were selected following the PICO model: Population (P: subjects with AT and CU), Intervention (I: treatment with LT4), Comparison (C: same patients before and after treatment/usual care or placebo), Outcome (O: signs and symptoms of CU). Randomized controlled trials (RCTs), retrospective and prospective cohort studies, and uncontrolled studies were included. No restrictions on language were applied.

### 2.3. Data Extraction and Quality Assessment

The following data were collected: first author, year, country, study design, number of subjects, type of controls, additional therapies, gender distribution, age, thyroid function, mean LT4 dose, method for outcome assessment, and number/percentage of patients undergoing clinical remission. Data extraction was performed independently by two authors (G.S. and A.C.) and their work was verified by a third author (G.B.).

The quality of evidence (QoE) was assessed using the Newcastle-Ottawa Scale (NOS) for case–control and cohort studies [13], the Version 2 of the Cochrane risk-of-bias tool (RoB 2) for RCTs, and the methodological index for nonrandomized studies (MINORS) tool for uncontrolled studies [14]. Two authors (G.S. and A.C.) independently performed the QoE, and any disagreements were resolved through discussion.

### 2.4. Statistical Analysis

The analysis was performed using RevMan software v. 5.4.1 (Cochrane Collaboration, Oxford, UK) and Comprehensive Meta-Analysis v. 3.7 (Biostat Inc., Englewood, NJ, USA). Mean difference (MD) or standardized mean difference (sMD) and 95% confidence intervals (Cis) were calculated to compare outcome measures after treatment. *I*^2^ statistic was applied to explore heterogeneity, with *I*^2^ > 50% and *p* < 0.1 indicating high between-study heterogeneity. If significant heterogeneity emerged, meta-analysis was performed using a random-effects model. Otherwise, a fixed-effects model was used. If more than one longitudinal measurement was available, the longest follow-up from each study was selected. Publication bias was assessed by funnel plot asymmetry as well as Egger’s test. Sensitivity analysis (omitting each single study to explore its effect on the overall meta-analysis) and subgroup analysis were conducted. Statistical significance was set at 0.05.

## 3. Results

**Figure 1:**
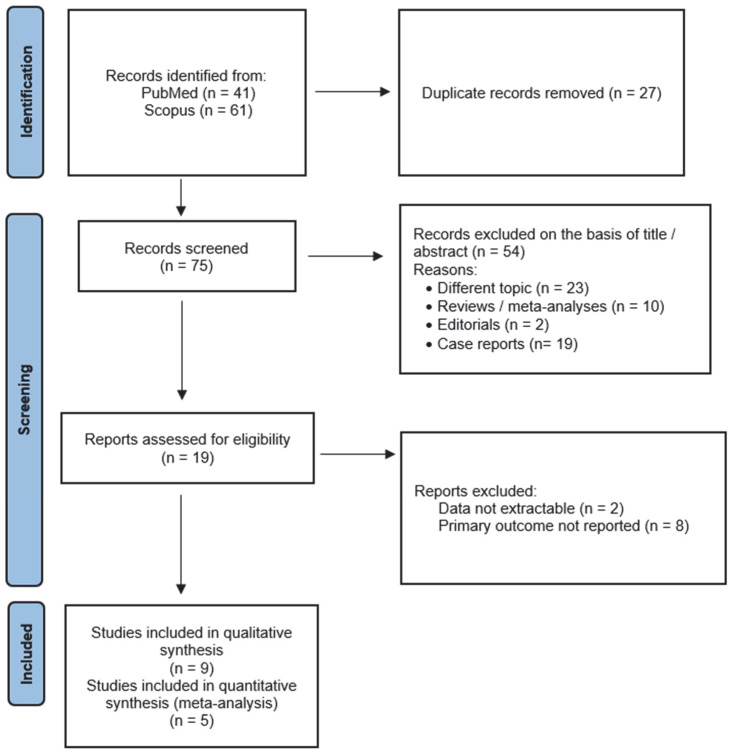
Preferred Reporting Items for Systematic reviews and Meta-Analysis (PRISMA) flowchart.

Using the above-mentioned search strategy, 102 abstracts were extracted. After the removal of 27 duplicates, 75 articles were screened. Of these, 54 were identified by title or abstracts as papers on other topics, review articles, editorials or case reports. Of the remaining 19 full-text articles assessed for eligibility, 9 [9,11,15,16,17,18,19,20,21] were included for qualitative synthesis and 5 [11,15,16,20,21] for quantitative synthesis (meta-analysis) (Figure 1, Table 1 and Table 2).

### 3.1. Quality of Evidence and Risk of Bias Assessment

Six [9,11,15,16,18,20] of the included studies had a prospective design and three [17,19,21] were retrospective. Four [9,15,16,21] had a control group and five [9,11,17,19,20] were uncontrolled. The study by Najafipour et al. had a randomized, placebo-controlled design [18]. Control groups were mainly composed of patients affected by AT not treated with LT4 and patients with CU treated with standard therapies. Quality of the evidence was high only in three studies [15,16,21], while the others ranged from moderate to poor quality. In addition, the only RCT [18] included in the review had a high risk of bias (Table 3).

### 3.2. Qualitative Synthesis of the Included Studies

Most studies included both patients with normal thyroid function and hypothyroidism. Treatment with LT4 had been prescribed for replacement therapy (aimed at normalizing TSH levels) or suppression therapy (aimed at lowering TSH levels and autoantibodies). In six studies [9,16,18,19,20,21], LT4 was used in combination with standard CU treatments. Sample sizes of treatment groups ranged from 8 to 46 patients. Follow-up lengths ranged from 6 weeks to 6 months. Clinical response to LT4 treatment was judged with heterogeneous symptom scores evaluating pruritus, lesions, itch, erythema, wheals, hives, sleep quality, and angioedema. Data on complete clinical remission was reported in one study [19], while the others [9,11,15,16,17,18,20,21] only described variations in symptoms score results. Only two studies [16,20] reported biochemical parameters as response, the neopterine and cytokine levels, respectively. Studies publication years ranged from 1989 to 2018, with only two studies published after 2012 (Table 4).

In a first retrospective uncontrolled study, Leznoff et al. reported data on 46 patients affected by AT (with or without hypothyroidism) and CU. LT4 treatment had a substitutive or suppressive purpose on TSH levels; the therapy was assumed with concomitant medications such as antihistamines, theophylline, a-agonists, and doxepin. The follow-up length was one month and data on thyroid hormones levels and LT4 treatment doses were not reported. Clinical remission of CU was reported in six patients (13%) [19].

In 1995, Rumbyrt et al. [9] conducted a prospective uncontrolled study on 10 patients with regular thyroid hormones values (two of them under LT4 substitution) undergoing LT4 suppression therapy in association with various standard CU treatments. After 3 months, seven patients (70%) showed clinical improvement, with decrease in frequency and severity of hives and need of standard medications to control symptoms [9].

Karaayvaz et al., in 2002 [15], evaluated prospectively 60 patients with AT and CU. Thirty subjects were treated with LT4 and 30 with ketotifen. A symptom score evaluating lesions, itching and angio-oedema was used to assess clinical response. Twenty-one patients were responsive to LT4 with a reduction of symptoms score > 50% and nine were nonresponsive (reduction < 50%). The authors also reported that responders had higher pretreatment autoantibodies and TSH mean levels (9.46 vs. 1.84 mcU/mL) [15].

In a prospective uncontrolled study, Aversano et al. treated 20 patients affected by CU and AT with LT4 to suppress TSH levels (target < 0.3 mcU/mL). Patients were euthyroid or affected by hypothyroidism. Mean pre- and post-treatment TSH levels were 5.29 and 0.17 mcU/mL, respectively. Clinical response was evaluated with a composed symptoms score assessing erythema, wheals, itching, and interference with sleep. A decrease in CU symptoms was observed in 16 patients after 3 months [11].

Kiyici et al. in 2010 [16] conducted a 3-month prospective controlled study on eight patients affected by AT and CU treated with LT4 and desloratadine compared with seven patients treated with desloratadine alone. Patients were euthyroid at baseline and the treatment had a suppressive aim with target TSH < 0.5 mcU/mL. Mean pre- and post-LT4 TSH were 1.73 and 0.52 mcU/mL with a mean treatment dose of 137 mcg/daily. Main outcome evaluated was Urticaria Activity Score (UAS). In both groups symptoms improved in comparison to baseline but no significative differences were found between groups [16].

Gulec et al. in 2011 [20] evaluated prospectively 27 patients with AT and CU treated with a standard LT4 dose of 100 mcg/daily. Data on pre and post treatment thyroid function were not available. A significant improvement in symptom score evaluating urticaria lesions (plaques), frequency of urticaria attacks, and intensity of itching and a decrease in neopterine levels were observed at 6 weeks. Notably, concomitant hydroxyzine was administered during the first week [20].

In 2012, Magen et al. [21]—in a retrospective study—compared 44 hypothyroid patients with AT and CU treated with LT4 + antihistamines and 44 euthyroid patients affected by CU without AT treated with antihistamines. An improvement in symptoms evaluated with UAS was observed at months 3 and 6 without significant differences between treatment and control groups [21].

Kim et al. in 2016 retrospectively evaluated 10 patients with AT and CU treated with LT4 until normalization of thyroid function, observing a reduction in spontaneous wheals or need of medications in 2 patients [17].

Finally, in 2018 Najafipour et al. [18] carried out a prospective randomized controlled study in which 36 patients were assigned to LT4 group (treatment group) and 36 to placebo group (control group). All patients were also treated with loratadine. At the 3-month follow-up, both groups had an improvement in itching and pruritus. However, in the treatment group, the recovery was faster and there were more patients without itching at 3 months. No data on thyroid function after treatments was reported [18].

### 3.3. Quantitative Analysis of the Included Studies

Since different symptom scales were used among the studies (Table 3), the extent of the changes was calculated using the sMD between groups. In the study by Magen et al. [21], the results were reported separately for subjects with positive and negative autologous serum skin test (ASST), so data were included as Magen 2012a (ASST+) and Magen 2012b (ASST−).

#### 3.3.1. Before–After Analysis

**Figure 2:**
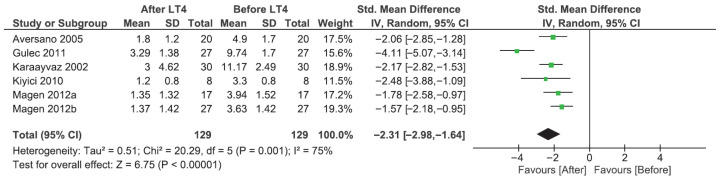
Effects of levothyroxine (LT4) administration on chronic urticaria (CU) clinical activity score. The studies of Aversano et al. [11], Gulec et al. [20]; Karaayvaz et al. [15], Kiyici et al. [16], and Magen et al. [21] were included.

**Figure 3:**
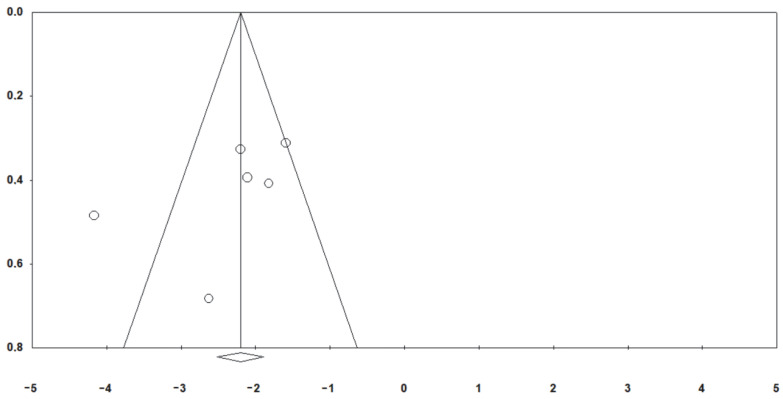
Funnel plot of the included studies.

According to the results of five studies [11,15,16,20,21], the treatment with LT4 was associated with a significant improvement in CU symptoms (sMD −2.31, 95% CI −2.98 to −1.64; *p* < 0.00001) (Figure 2). Heterogeneity between studies was high (*I*^2^ = 75%, *p* = 0.001) and decreased to 0% when the study by Gulec et al. was removed, but the results remained significant (sMD −1.92, 95% CI −2.26 to −1.58; *p* < 0.00001). No significant publication bias emerged (Egger’s test *p* = 0.238, Figure 3).

**Figure 4:**
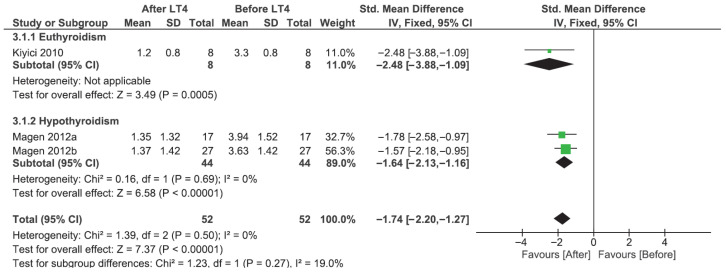
Effects of L-thyroxine (LT4) administration on chronic urticaria (CU) clinical activity score in subjects with normal thyroid function and hypothyroidism. The studies of Kiyici et al. [16] and Magen et al. [21] were included.

A subgroup analysis was performed to investigate the main source of heterogeneity. First, a comparison was made between treatment with LT4 for subjects with regular thyroid function and hypothyroidism. According to the results of our analysis, subjects with normal thyroid function showed a higher improvement in CU symptom scores, but the difference between groups was not statistically significant (*p* = 0.27) (Figure 4).

**Figure 5:**
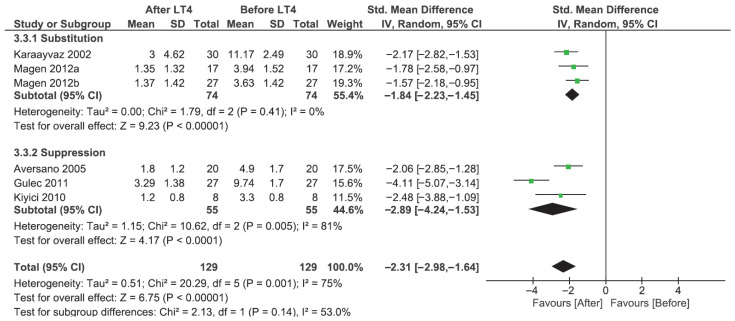
Effects of suppressive or substitutive levothyroxine (LT4) administration on chronic urticaria (CU) clinical activity score. The studies of Aversano et al. [11], Gulec et al. [20]; Karaayvaz et al. [15], Kyici et al. [16], and Magen et al. [21] were included.

We therefore compared subjects undergoing suppression therapy and thyroid hormone replacement. As already observed for subjects with normal thyroid function, the effect of LT4 on CU symptoms was more relevant in subjects undergoing suppressive therapy, but the difference between subgroups was not significant (*p* = 0.14) (Figure 5).

#### 3.3.2. Comparison Analysis

**Figure 6:**
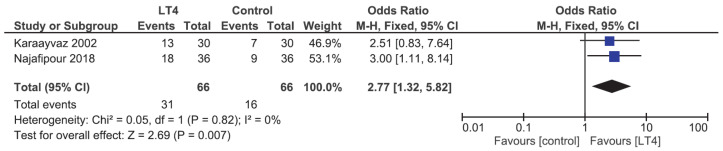
Comparison between levothyroxine (LT4) or placebo/standard care administration on positive response to treatment. The studies of Karaayvaz et al. [15] and Najafipour et al. [18] were included.

**Figure 7:**
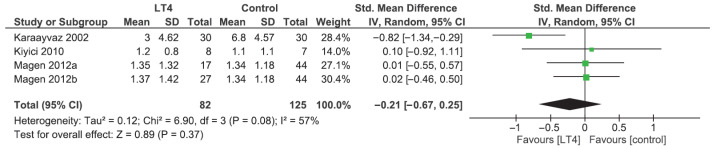
Comparison between levothyroxine (LT4) or placebo/standard care administration on chronic urticaria (CU) clinical activity score. The studies of Karaayvaz et al. [15], Kiyici et al. [16], and Magen et al. [21] were included.

Finally, we performed a comparison analysis between subjects treated with LT4 and subjects treated with placebo or usual standard care. Although treatment with LT4 is associated with a higher likelihood of a positive response (OR 2.77, 95% CI 1.32 to 5.82; *p* = 0.007) (Figure 6), its effect does not appear to be superior to that of placebo or standard therapy on clinical measures of disease activity (*p* = 0.37) (Figure 7).

## 4. Discussion

### Clinical Outcomes Concerning the Effects of LT4 Treatment in CU Patients

**Figure 8:**
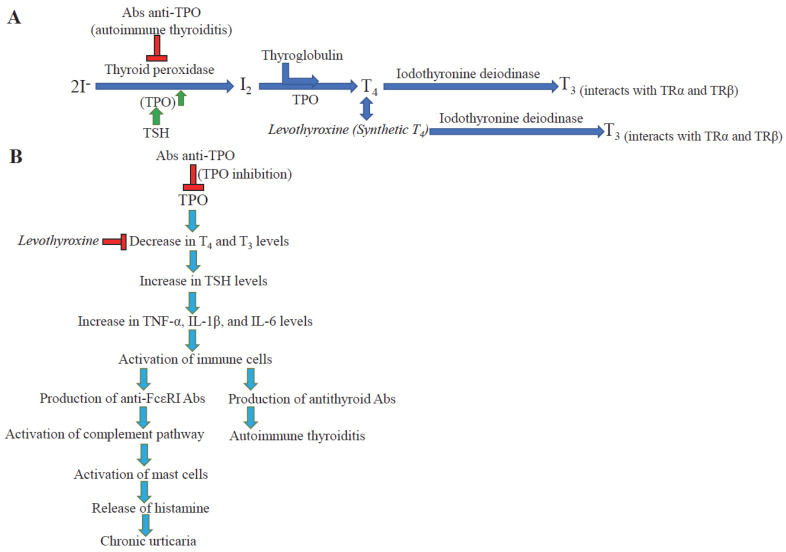
Pathogenetic relationship between AT and CU. (**A**) Biochemical pathways related to the synthesis of T4 and T3 thyroid hormones and the biological effects of anti-TPO antibodies and the drug Levothyroxine. (**B**) Molecular pathways that describe the relationship between the pathogenetic mechanisms of AT and CU. The biological effects of anti-TPO antibodies and the drug Levothyroxine are also reported. AT: autoimmune thyroiditis; CU: chronic urticarial; FCεRI: Fc epsilon Receptor I; IL-1β: Interleukin-1β; IL-6: Interleukin-6; TPO: Thyroid peroxidase; TSH: Thyroid-Stimulating Hormone; TRα: Thyroid Hormone Receptor alpha; TRβ: Thyroid Hormone Receptor beta; TNF-α: Tumor necrosis factor-α. Blue/light blue arrows: activation; red arrows: block.

The evidence on the effects of LT4 on CU in patients with AT is still very limited and controversial. At first glance, the present systematic review has shown a potential benefit of LT4 in CU, as reflected by the pre-post analysis; indeed, 68 out of 152 subjects (45%) exhibited positive effects after treatment. This could be in agreement with the proposed role of TSH as immunomodulatory agent [11]; indeed, treatment with LT4 can directly lower TSH levels, and possibly suppress its inflammatory action on target organs as the skin. On the other hand, a small uncontrolled study involving 10 euthyroid patients reported that seven subjects with elevated anti-thyroid antibodies experienced complete resolution of CU symptoms within one month of LT4 suppressive therapy, whereas the three patients with undetectable antibodies showed no clinical response [9]. Despite its small sample size and lack of a control group, these results suggest a potential link between TA and CU. The ability of LT4 to decrease anti-thyroid antibodies has already been reported, and the mechanism likely involves TSH suppression [22], thus further corroborating the hypothesis that TSH normalization is the main effector in the resolution of CU symptoms. Interestingly, it was reported in a study that TPO-antibodies were detected in 30% of patients with CU [18]. TPO in an enzyme that plays a pivotal role in the hormonogenesis in the thyroid and functions also as a thyroid differentiation marker and as autoantigen, indicated as microsomal antigen [23]. TPO catalyzes the oxidation of iodide that is necessary for the iodination of the thyroglobulin tyrosyl residues and plays a pivotal role in the process of oxidative coupling of hormogenic iodotyrosine residues into the hormone T4 (Figure 8A) [23]. Notably, T4 can be deiodinated into the hormone T3 due to the presence and catalytic activity of the enzyme iodothyronine deiodinase (Figure 8A) [23]. It was reported that T3 can interact with thyroid hormone nuclear receptors α and β, regulating the gene transcription of specific targets [23]. Several reports indicate that the activity of TPO is modulated by TSH [23,24], demonstrating the central and fundamental role of this molecular marker in the physiological mechanisms of the thyroid functions. Noteworthy, the presence of anti-TPO antibodies in the serum of patients determines the inhibition of the activity of this enzyme (Figure 8A) and, consequently, the reduction of the synthesis of thyroid hormones, contributing to the development of AT [6]. Treatment of AT involves replacing thyroid hormone deficiency with the use of the synthetic hormone LT4 (Figure 8A), which can compensate for reduced levels of endogenous T4 [25]. Interestingly, there is strong clinical evidence indicating that levels of IgG anti-TPO are more often elevated in CU than those of other thyroid antibodies, suggesting the existence of a molecular network between AT and CU [6].

Notably, the presence in the serum of the patients of anti-TPO antibodies determines the inhibition of TPO activity, which leads to a decrease in T4 and T3 levels (Figure 8B). This decrease is compensated by the treatment with LT4 (Figure 8B). A reduction in the levels of T4 and T3 hormones determines an increase in TSH levels, which is associated with augmented levels of several pro-inflammatory molecules (Figure 8B), such as TNF-α, IL-1β, and IL-6 [26]. These pro-inflammatory markers can induce a cascade of molecular signals that activate the immune cells of the patients, determining the production of several anti-thyroid antibodies (such as anti-TPO and anti-thyroglobulin) (Figure 8B) and the worsening of the AT pathological condition [6]. Interestingly, the TNF-α-, IL-1β-, IL-6-mediated activation of the immune cells determines also an increase in the levels of anti-FcεRI antibodies (Figure 8B), which play a pivotal role in the pathogenesis of CU [6,26]. These antibodies can activate the classical complement pathway (Figure 8B), generating anaphylatoxins such as C5a, which can bind to its receptor in mast cells, inducing their activation and degranulation [6]. The activated mast cells release high levels of histamine (Figure 8B), which triggers vasodilation, increased vascular permeability, and activation of nerve endings, leading to the development of CU [26]. This scientific evidence support the hypothesis that there could be a molecular and cellular network linking the pathogenesis of CU to the pathogenesis of AT and could suggest a therapeutic approach based on LT4 treatment in patients affected by CU [6,18,26]. However, the proposed molecular network (Figure 8) is speculative and requires confirmation in future mechanistic studies. Indeed, treatment effects appear to be highly heterogeneous and unpredictable across patients, suggesting that the underlying mechanisms are not yet fully understood. This variability may suggest that additional molecular pathways contributing to the amelioration of CU symptoms with LT4 therapy are yet to be elucidated.

Data from the literature show that the efficacy of this therapy seems to differ between hypothyroid and euthyroid patients. In hypothyroid patients, LT4 is indicated for thyroid replacement but does not have a clear effect in the improvement in CU outcomes [6,27]. In euthyroid patients, the role of LT4 therapy is even more controversial, with some case series showing some benefit but no high-quality controlled trials to warrant its routine use in the clinical setting [27]. This is supported by a recent systematic review of the literature by Kolkhir and colleagues, which reported improvement or remission of CU in 28% of patients with hypothyroidism and in 67% of patients with normal thyroid function [6].

Interestingly, our subgroup analysis did not show significant differences between euthyroid and hypothyroid subjects. This may suggest that the therapeutic effect of LT4 is not dependent on overt thyroid dysfunction or that patients with hypothyroidism have other underlying unexplained effectors contributing to the pathogenesis and worsening of CU. This hypothesis is also supported by the observation that patients with hyperthyroidism and CU show beneficial effects on CU after normalization of thyroid function with methimazole or propylthiouracil therapy [6].

Similarly, no differences emerged when comparing replacement versus suppressive LT4 therapy. Although TSH suppression could hypothetically reduce autoimmune activation or inflammatory pathway activation [11], the analysis of the available evidence is inconclusive. However, several confounding factors need to be accounted for, including dose variability, treatment duration, patient selection and other used medications directly targeting CU. On the other hand, iatrogenic subclinical hyperthyroidism is associated with an increased risk of osteoporosis [28], atrial fibrillation [29], and ischemic heart disease [30]; therefore, the unclear benefits of suppressive therapy should be weighed against concomitant comorbidities.

A layer of complexity is also added when comparing treatment with control groups. In fact, the clinical improvement observed in the before-and-after analysis was not found to be superior when LT4 therapy was compared with placebo or standard of care. In light of this, the reported improvement may be either related to treatment, to the natural history of CU disease or other concomitant therapies. Indeed, other studies that report a significant improvement in CU outcomes in patients treated with LT4 display the same progresses in the untreated euthyroid controls [16,21]. In addition, pooled data from studies with heterogeneous designs should be interpreted with caution. More controlled studies are therefore required to have a better understanding of the potential outcomes of LT4 treatment on CU.

The analyzed studies employed a variety of methods and scales to evaluate patient outcomes. Only two groups assessed improvements in CU symptoms using the guideline-recommended UAS, a patient-reported outcome measuring tool. This depicts symptom severity by rating the extent of wheals and both daytime and nocturnal pruritus on a scale from 0 to 3 (0 = absent, 1 = mild, 2 = moderate, 3 = severe) [18,19]. Other authors used 12- [11], 15- [20], and 16- [17] point scales for symptoms, or visual analog scales for itching [9], that were poorly comparable. Another study defined the efficacy of LT4 treatment as either the complete resolution of daily spontaneous wheals, or a reduction in the need for medication to achieve symptom control [17]. Only one study objectively assessed the extent and severity of urticaria plaques [20], and only two studies introduced biochemical parameters as means of assessment of LT4 treatment efficacy (neopterine and cytokine) [19,20]. This heterogeneity between studies is challenging to interpret. Despite being a universal and guideline-suggested tool, the reliance on subjective assessment through the UAS introduces potential sources of bias, including inter-individual variability in symptom awareness and reporting, and a possible “placebo effect” as well. Even the more objective assessment of urticaria plaques or biochemical parameters is not standardized and lacks clinical evidence. These factors can heavily complicate the evaluation of treatment efficacy and there is a clear need for the implementation of more objective and standardized ways for assessing CU signs and symptoms.

It should be noted that our study has some important limitations, which are primarily those found in the current literature. In fact, there are few available studies, they have very small sample sizes, and no studies published between 2018 and 2025 met the inclusion criteria. This significant gap in the literature of recent years undoubtedly influences our analysis. Moreover, the quality of the included studies ranged from moderate to poor, with only a few high-quality studies, and the only randomized controlled trial was judged to be at high risk of bias. In addition, although the clinical course of CU in response to the decline in antibody titers observed during LT4 therapy deserved investigation, data on thyroid function and levels of autoantibodies after treatment were not sufficient to be analyzed.

## 5. Conclusions

Although the potential positive effect of LT4 treatment on CU symptoms was suggested in some studies, data available in the literature is too heterogeneous to reach strong conclusions, and the improvement in symptoms after starting LT4 therapy was not confirmed when compared with a placebo or standard of care. Low sample sizes, lack in characterization of treatment and control groups, and different outcomes and purposes of LT4 treatment represent further limitations that prevent LT4 therapy from being considered a potential remedy for CU in individuals with AT. Further studies with stronger designs are needed to properly evaluate the role of LT4 in CU treatment and to improve and deepen the knowledge of the molecular pathways and markers that link CU and AT.

## Figures and Tables

**Table 1:** Design of the included studies.

First Author	Year	Country	Study Design	Type of Controls	Subjects Treated (n, %)	Mean Age ± SD	Gender (M/F)
Aversano [11]	2005	Italy	Prospective uncontrolled	-	20 (100%)	36.8 ± 11.9	0/20
Gulec [20]	2011	Türkiye	Prospective uncontrolled	-	27 (100%)	35.7 ± 10.86	7/20
Karaayvaz [15]	2002	Türkiye	Prospective controlled	Sex-, age-, and symptoms-matched subjects treated with ketotifen	30 (50%)	R 37.29 ± 11.95 NR 37.56 ± 13.51	6/24
Kim [17]	2016	Korea	Retrospective uncontrolled	-	10 (5.4%)	-	1/9
Kiyici [16]	2010	Türkiye	Prospective controlled	Desloratadine alone	8 (53.3%)	37.9 ± 14.7 (all)	1/14 (all)
Leznoff [19]	1989	Canada	Retrospective, uncontrolled	-	46 (100%)	-	-
Magen [21]	2012	Israel	Retrospective controlled	Euthyroid subjects with urticaria under treatment with antihistamines	44 (50%)	ASST+ 45.2 ± 7.1 ASST− 43.9 ± 9.4	3/41
Najafipour [18]	2018	Iran	Prospective randomized placebo-controlled	AT with urticaria under treatment with antihistamines (Loratadine) and placebo	36 (50%)	32.3 ± 9.99	2/34
Rumbyrt [9]	1995	USA	Prospective uncontrolled	-	10 (100%)	43	3/7

ASST = autologous serum skin test; AT = autoimmune thyroiditis; NR = non-responders; R = responders.

**Table 2:** Characteristics of the included studies.

First Author	Thyroid Status	Subjects Treated (n, %)	Purpose (Target)	Additional Therapy	Follow-Up (Months)	Outcome Assessment
Aversano [11]	12 Euthyroidism 8 Hypothyroidism	20 (100%)	Suppression (TSH < 0.3 mcIU/mL)	-	3	Clinical score 0–12: erythema, wheals, itching, interference with sleep.
Gulec [20]	Not stated	27 (100%)	Suppression (target not stated)	Hydroxyzine (first week only)	1.5	Clinical score 0–15: number of urticaria lesions, size of lesions, duration of lesions, frequency of urticaria attacks and intensity of itching.
Karaayvaz [15]	53 Euthyroidism 6 Subclinic hypothyroidism 1 Hypothyroidism	30 (50%)	Substitution	-	1, 1.5, and 2	Symptoms score 0–16 (no. Lesions, duration lesions, itching, angio-oedema) + % change score 100% (complete). <50% (unsuccessful) e ≥ 50% (partial).
Kim [17]	158 Euthyroidism 8 Hyperthyroidism 8 Subclinic hypothyroidism 10 Hypothyroidism	10 (5.4%)	Substitution	-	Unspecified	Elimination of daily spontaneous wheals or a significant reduction of medication to control daily spontaneous wheals
Kiyici [16]	15 Euthyroidism	8 (53.3%)	Suppression (TSH < 0.5 mcIU/mL)	Desloratadine	Unspecified	UAS: pruritus (0–3) and wheals (size 0–3 + number 0–3) + serum cytokines.
Leznoff [19]	Not stated	46 (100%)	Suppression or substitution	Antihistamines, theophylline, α-agonists, ketotifen, doxepin, and/or prednisone	3	Unspecified.
Magen [21]	44 Euthyroidism 44 Hypothyroidism	44 (50%)	Substitution (TSH < 4.5 mcIU/mL)	Antihistamines	1	UAS: 0–12.
Najafipour [18]	Not stated	36 (50%)	Suppression	Loratadine	3, 6	The severity of itching was evaluated according to the visual analog scale before and after therapy.
Rumbyrt [9]	8 Euthyroidism 2 Hypothyroidism	10 (100%)	Suppression	Hydroxyzine, cyproheptadine, ranitidine, diphenhydramine, prednisone, H1, H2 blockers, hydroxyzine, astemizole, doxepin, famotidine	3	Unspecified.

TSH = thyroid-stimulating hormone; UAS = Urticaria Activity Score.

**Table 3:** Quality assessment for the included studies.

Study	Tool	Q1	Q2	Q3	Q4	Q5	Q6	Q7	Q8	Score	Quality
Aversano [11]	MINORS	2	0	2	2	0	2	2	0	10	Moderate
Gulec [20]	MINORS	1	1	2	2	2	2	2	0	12	Moderate
Kim [17]	MINORS	1	2	1	2	0	2	2	0	10	Moderate
Leznoff [19]	MINORS	1	0	0	0	0	2	2	0	5	Poor
Rumbyrt [9]	MINORS	2	2	2	2	0	2	2	0	12	Moderate
Kiyici [16]	* NOS	★	★	★	★	★★	★	★	★	9	High
Karaayvaz [15]	* NOS	★	★	★	★	★★	★	★	★	9	High
Magen [21]	° NOS	★	★	★	★	★★		★	★	8	High
		**D1**	**D2**	**D3**	**D4**	**D5**					**Overall**
Najafipour [18]	RoB2	Some concerns	Some concerns	High risk	High risk	Some concerns					High risk

° NOS for case–control studies: Q1: Case definition; Q2: Representativeness of the cases; Q3: Selection of controls; Q4: Definition of controls; Q5: Comparability of cases and controls; Q6: Ascertainment of exposure; Q7: Same method of ascertainment; Q8: Nonresponse rate. Each ★ corresponds to one point. * NOS for cohort studies: Q1: Representativeness of the exposed cohort; Q2: Selection of the nonexposed cohort; Q3: Ascertainment of exposure; Q4: Demonstration that outcome of interest was not present at start of study; Q5: Comparability of cohorts; Q6: Assessment of outcome; Q7: Length of follow-up; Q8: Adequacy of follow-up of cohorts. Each ★ corresponds to one point. MINORS: Q1: A stated aim of the study; Q2: Inclusion of consecutive patients; Q3: Prospective collection of data; Q4: Endpoint appropriate to the study aim; Q5: Unbiased evaluation of endpoints; Q6: Follow-up period appropriate to the major endpoint; Q7: Loss to follow-up not exceeding 5%; Q8: prospective calculation of the study size). RoB2: D1: bias due to randomization process; D2: deviation from intended intervention; D3: missing outcome data; D4: measurement of outcomes; D5: selection of the reported results. MINORS, Methodological Index for Non-Randomized Studies; NOS, Newcastle-Ottawa Scale; RoB, risk of bias.

**Table 4:** Characteristics of the included studies (results).

First Author	Antibody-Positive Subjects (n, %)	Mean LT4 Dose (mcg/day)	Purpose (Target)	Outcome Assessment Results	Positive Response (n%)
Aversano [11]	20 (100%)	-	Suppression (TSH < 0.3 mcIU/mL)	Decrease in itching 2.15 ± 0.587 to 1 ± 0.324 (<0.0001); erythema 1.2 ± 0.447 to 0.2 ± 0.41 (*p* < 0.0001); wheals 0.35 ± 0.489 to 0 ± 0 (*p* = 0.0047); sleep 1.3 ± 0.571 to 0.6 ± 0.503 (<0.0001).	16 (80%)
Gulec [20]	27 (100%)	100	Suppression (target not stated)	Decrease in total score from 9.74 ± 1.70 to 3.29 ± 1.38 (*p* < 0.001).	-
Karaayvaz [15]	30 (100%)	100	Substitution	Total symptoms decreased from 11.17 ± 2.49 to 3.00 ± 4.62 (*p* < 0.001).	21 (70%)
Kim [17]	10 (100%)	-	Substitution	Elimination of daily spontaneous wheals or a significant reduction of medication to control daily spontaneous wheals	2 (20%)
Kiyici [16]	8 (100%)	137	Suppression (TSH < 0.5 mcIU/mL)	Pruritus decreased from 1.7 ± 0.4 to 0.5 ± 0.3 (*p* = 0.01) and wheal decreased from 1.6 ± 0.5 to 0.7 ± 0.6 (*p* = 0.01). Difference between LT4 and Desloratadine group was not significant (*p* = 0.46).	-
Leznoff [19]	46 (100%)	-	Suppression or substitution	Clinical remission (unspecified).	4 (8.7%)
Magen [21]	44 (100%)	-	Substitution (TSH < 4.5 mIU/L)	Significant decrease in UAS after 3 and 6 months in all the groups without significant differences between subject with or without hypothyroidism.	-
Najafipour [18]	36 (100%)	50	Suppression	Itching in the mild, moderate, and severe degrees were 12, 18, and 6, respectively in the case group, and, after treatment, non-itching point was observed in 18 patients.	18 (50%)
Rumbyrt [9]	7 (70%)	111.2	Suppression	Improvement in symptoms (unspecified).	7 (100%)

TSH = thyroid-stimulating hormone; UAS = Urticaria Activity Score.

## Data Availability

No new data were created or analyzed in this study. Data sharing is not applicable to this article.

## Supplementary Materials

The following supporting information can be downloaded at: https://www.mdpi.com/article/10.3390/ijms27146348/s1.

## Abbreviations

The following abbreviations are used in this manuscript:

AT	Autoimmune thyroiditis
CI	Confidence interval
CU	Chronic urticaria
FCεRI	Fc epsilon Receptor I
IL-1β	Interleukin-1β
IL-6	Interleukin-6
LT4	L-thyroxine
MD	Mean difference
NOS	Newcastle-Ottawa Scale
QoE	Quality of evidence
RCTs	Randomized controlled trials
RoB 2	Cochrane risk-of-bias tool 2
sMD	Standardized mean difference
TNF-α	Tumor necrosis factor-α
TPO	Thyroid peroxidase
TRα	Thyroid Hormone Receptor alpha
TRβ	Thyroid Hormone Receptor beta
TSH	Thyroid-stimulating hormone
MINORS	Methodological index for nonrandomized studies
UAS	Urticaria Activity Score
